# Intronic miR-744 Inhibits Glioblastoma Migration by Functionally Antagonizing Its Host Gene MAP2K4

**DOI:** 10.3390/cancers10110400

**Published:** 2018-10-25

**Authors:** Max Hübner, Christian Ludwig Hinske, David Effinger, Tingting Wu, Niklas Thon, Friedrich-Wilhelm Kreth, Simone Kreth

**Affiliations:** 1 Department of Anesthesiology, University Hospital, LMU Munich, 81377 Munich, Germany; max.huebner@med.uni-muenchen.de (M.H.); christian.hinske@med.uni-muenchen.de (C.L.H.); david.effinger@med.uni-muenchen.de (D.E.); Tingting.wu@med.uni-muenchen.de (T.W.); 2 Walter-Brendel Center of Experimental Medicine, Faculty of Medicine, LMU Munich, 81377 Munich, Germany; 3 Department of Neurosurgery, University Hospital, LMU Munich, 81377 Munich, Germany; Niklas.Thon@med.uni-muenchen.de (N.T.); friedrich-wilhelm.kreth@med.uni-muenchen.de (F.-W.K.)

**Keywords:** glioblastoma, migration, microRNAs, MAP2K4

## Abstract

Background: The second intron of Mitogen-Activated Protein Kinase Kinase 4 (MAP2K4), an important hub in the pro-invasive MAPK pathway, harbors miR-744. There is accumulating evidence that intronic micro-RNAs (miRNAs) are capable of either supporting or restraining functional pathways of their host genes, thereby creating intricate regulative networks. We thus hypothesized that miR-744 regulates glioma migration by interacting with its host’s pathways. Methods: Patients’ tumor specimens were obtained stereotactically. MiR-744 was overexpressed in U87, T98G, and primary glioblastoma (GBM) cell lines. Cell mobility was studied using migration and Boyden chamber assays. Protein and mRNA expression was quantified by SDS-PAGE and qRT-PCR. Interactions of miR-744 and 3’UTRs were analyzed by luciferase reporter assays, and SMAD2/3, p38, and beta-Catenin activities by TOP/FOPflash reporter gene assays. Results: As compared to a normal brain, miR-744 levels were dramatically decreased in GBM samples and in primary GBM cell lines. Astrocytoma WHO grade II/III exhibited intermediate expression levels. Re-expression of miR-744 in U87, T98G, and primary GBM cell lines induced focal growth and impaired cell mobility. Luciferase activity of 3’UTR reporter constructs revealed the pro-invasive factors TGFB1 and DVL2 as direct targets of miR-744. Re-expression of miR-744 reduced levels of TGFB1, DVL2, and the host MAP2K4, and mitigated activity of TGFB1 and DVL2 downstream targets SMAD2/3 and beta-Catenin. TGFB1 knock-down repressed MAP2K4 expression. Conclusion: MiR-744 acts as an intrinsic brake on its host. It impedes MAP2K4 functional pathways through simultaneously targeting SMAD-, beta-Catenin, and MAPK signaling networks, thereby strongly mitigating pro-migratory effects of MAP2K4. MiR-744 is strongly repressed in glioma, and its re-expression might attenuate tumor invasiveness.

## 1. Introduction

Micro-RNAs (miRNAs) are short RNA molecules with an established role as important epigenetic regulators of the transcriptome via recognition of base-complementary signals in the 3’ untranslated regions of target mRNAs [1,2]. Interestingly, the majority of human miRNA genes are located within non-coding regions of protein-coding genes [3,4]. It also appears that this colocalization leads to coregulation in several instances, either through cotranscription and therefore coexpression, or via shared cis-regulatory elements [5]. Increasing evidence has begun to unravel the shades of a mechanism bearing an important role in the regulation of central cellular pathways, rather than just being a biological pendent of an information compression algorithm. However, we are only on the verge of understanding its impact on health and disease [6,7]. The Mitogen-Activated Protein Kinase (MAPK) pathway is one of such central signal transduction pathways, regulating a plethora of essential cellular functions, including proliferation, differentiation, and modulation of gene expression. One of its members, Mitogen-Activated Protein Kinase Kinase 4 (MAP2K4), is a well-known tumorigenic kinase with an established role in metastasis, invasion, and cancer progression [8,9,10]. *MAP2K4* in turn hosts the intronic microRNA *hsa-miR-744*, located in the second intron of its host. Even though MAP2K4 has long been recognized as a potent proto-oncogene, little is known about its intronic miRNA. Most importantly, the relationship between MAP2K4 and miR-744 is as yet completely uncharacterized. Due to the central and established role of MAP2K4 in tumor biology [11,12], we hypothesized miR-744 to be guilty by association, possibly augmenting or antagonizing MAP2K4s tumor-promoting effects.

In the following manuscript, we provide new and unprecedented evidence of a significant role of miR-744 in the regulation of its host gene MAP2K4 in human glioma. This is accomplished via controlling both expression and functional aspects of its host gene. We show how miR-744 inhibits cell migration and invasion, a key characteristic of glioblastoma (GBM), via targeting three essential cellular pathways. We finally validate our hypothesis in cell line experiments as well as patients´ tissue samples. We believe that our findings shed some more light on the as yet blurry contours of the mechanisms underlying tumor development through dysregulation of cellular signaling, and portray miR-744 as a central molecule in the formation of GBM.

## 2. Results

### 2.1. miR-744 Is Strongly Repressed in Human Glioma

**Figure 1:**
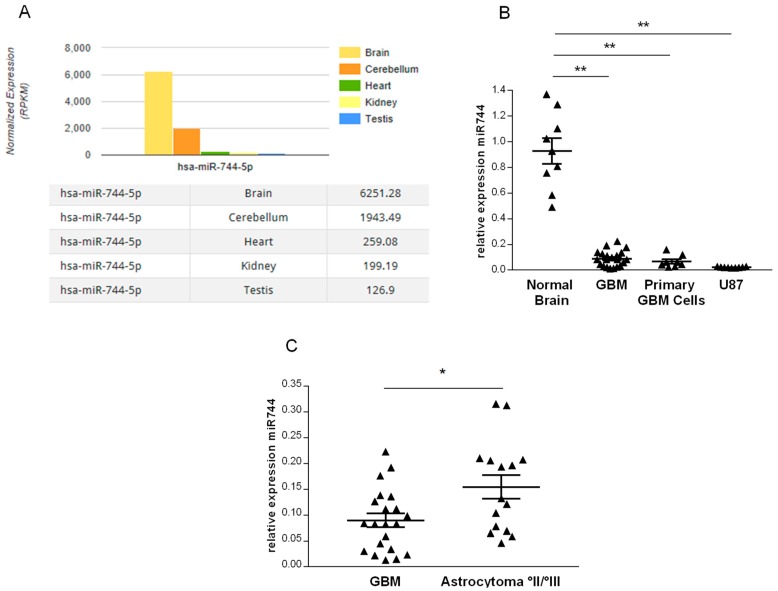
miR-744 is strongly repressed in glioma. MiR-744 expression was quantified by qRT-PCR. U47 served as the endogenous reference. (**A**) Expression levels of miR-744 in five different tissues. (**B**) Expression of miR-744 in normal brain tissue (NB) (n = 9), glioblastoma (GBM) (n = 21), primary GBM cell lines (n = 9), and U87 cells (n = 9), *p* < 0.001. (**C**) Expression of miR-744 in WHO grade °II/°III glioma (n = 15) compared to GBM (n = 21), *p* = 0.034. ** *p* < 0.001; * *p* < 0.05.

To identify human tissues in which miR-744 may fulfill important regulatory functions, we used the intragenic microRNA database miRIAD (http://bmi.ana.med.uni-muenchen.de/miriad/) [4], and screened for tissues with high expression levels of this miRNA. As depicted in Figure 1A, among five different tissues deposited in miRIAD (heart, testis, kidney, cerebellum, and brain), human brain exhibited the highest expression levels of miR-744, which suggests its implication in the maintenance of homeostatic conditions in the central nervous system. To investigate our initial hypothesis, we next quantified miR-744 in stereotactically obtained GBM specimens and primary cell lines obtained from open GBM resections by qRT-PCR, and found a dramatic reduction of miR-744 as compared to normal brain tissue (Figure 1B; GBM samples: reduction by 90.3% ± 14.7%, primary GBM cell lines: reduction by 92.7% ± 7.3%; n = 9 for normal brain tissue, n = 21 for GBM samples, n = 8 for primary GBM cell lines; *p* < 0.01). Also, we could detect reduced expression of miR-744 in U87 cells, a human GBM cell line (Figure 1B; reduction by 97.7% ± 6%, n = 9, *p* < 0.001).

Collectively, this data shows that miR-744 is highly expressed in human brain tissue, whereas it is almost entirely repressed in GBM.

To assess the expression of miR-744 in human glioma of different grades, we quantified miR-744 in 15 stereotactically obtained WHO II/III tumors by qRT-PCR. As depicted in Figure 1C, we found miR-744 also to be repressed; however, expression levels were significantly higher as compared to GBM (48% ± 20%; WHO II/III: n = 15, GBM: n = 21, *p* = 0.034). It thus appears that miR-744 expression is inversely correlated with tumor grade and may contribute to increased tumor aggressiveness.

### 2.2. Overexpression of miR-744 Reduces Migration of GBM Cells

**Figure 2:**
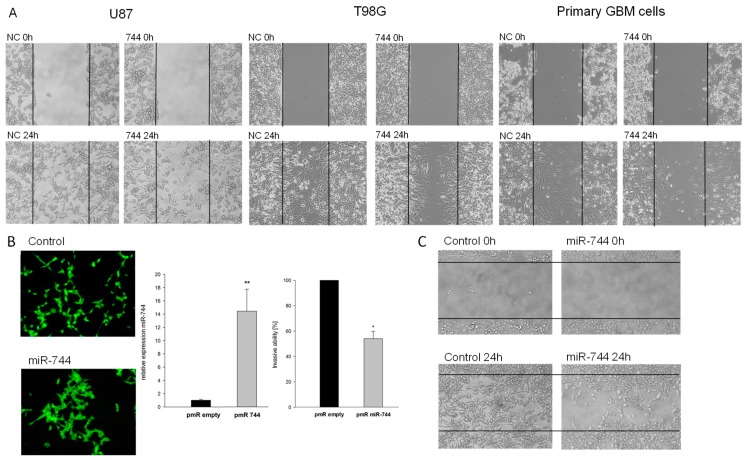
Overexpression of miR-744 induces focal cell growth and inhibits invasion and migration. (**A**) 2D migration assays of transiently miR-744 transfected cells (left panel: U87; middle panel: T98G; right panel: primary GBM cell lines) at start and after 24 h. Lines mark the initially cell-free area. A typical example of 3 experiments is shown. (**B**) Fluorescence microscopy of control and stably miR-744 overexpressing cells. (**C**) 2D migration assays of stably transfected cells; depicted are start state and after 24 h of incubation. Lines mark the cell-free area. A typical example of 3 similar experiments is shown. (**D**) Left panel: MiR-744 levels after stable transfection (induction 14.4-fold ± 6.0, n = 5, *p* < 0.001). Right panel: Invasion after stable transfection of U87 cells, measured with Collagen-coated Boyden Chamber invasion assays (n = 4, *p* = 0.029). ** *p* < 0.001; * *p* < 0.05.

It is a frequently occurring phenomenon that tumors down-regulate, or even hamper, the expression of genes that are not useful for malignant transformation. Our next aim was to understand the reasons for miR-744 downregulation in human GBM, and thus we investigated the biological functions of miR-744 in glioma cells. To this end, we transiently re-expressed miR-744 in U87, T98G, and primary GBM cell lines by transfection of the respective premiR and assessed the resulting phenotype. Surprisingly, we could not detect any alterations of apoptosis or proliferation after transfection of miR-744 (data not shown). 2D wound closure assays however, revealed a strong impact of miR-744 on cellular migration, which was markedly attenuated in miR-744 transfected cells (Figure 2A). To study the long-term effects of miR-744 on cellular migration, we constructed a miR-744 delivering expression vector, and stably transfected U87 GBM cells (Figure 2D, left panel). As shown in Figure 2B, overexpression of miR-744 leads to a decrease in cellular spreading and induces focal growth, pointing towards an alteration of cellular mobility. Importantly, 2D migration and Boyden Chamber assays revealed a less invasive phenotype (Figure 2C,D, right panel; reduction of 46% ± 5.8%, n = 4, *p* = 0.029). Taken together, this data shows that miR-744 inhibits migration of GBM cells.

### 2.3. TGFB1 and DVL2 are Direct Targets of miR-744, Regulate Migration in GBM Cells, and are Induced in Tissue of Human Malignant Glioma

**Figure 3:**
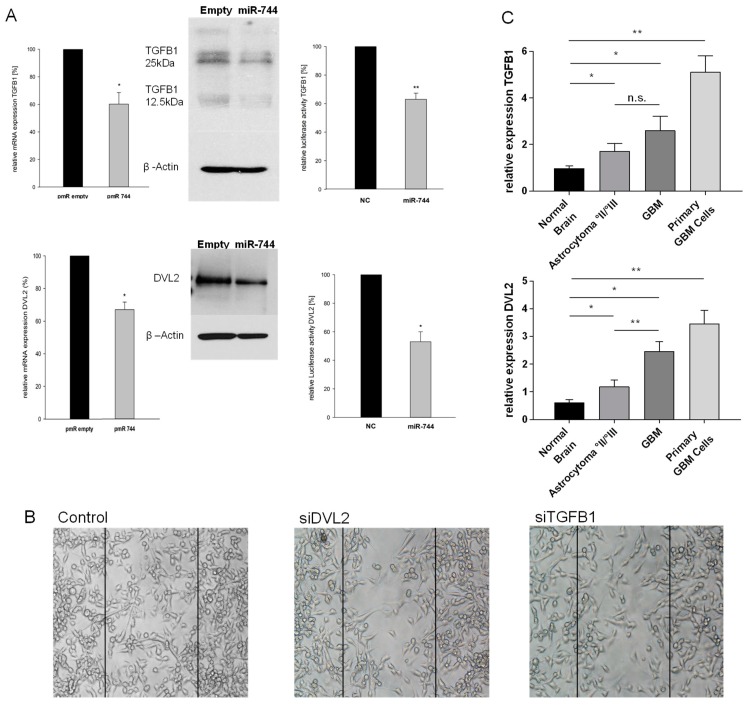
Transforming Growth Factor Beta 1 (TGFB1) and Dishevelled2 (DVL2) are direct targets of miR-744, regulate migration, and are induced in GBM and in Astrocytoma °II/°III. (**A**) Left and middle panels: mRNA and protein expression of TGFB1 and DVL2 in U87 cells stably transfected with miR-744 or empty vector control, respectively (n = 5, *p* < 0.05). Right panels: TGFB1 and DVL2 3’UTR Luciferase Reporter Gene activity after co-transfection of the respective reporter vectors with miR-744 or with scrambled control, respectively (DVL2: −47% ± 3,2%, TGFB1: −37% ± 2%, n = 5, *p* < 0.05). All Luciferase and mRNA experiments were performed in triplicates. (**B**) 2D migration assay after transient knock-down of DVL2 and TGFB1 in U87 cells. One representative example of 3 experiments is shown. (**C**) Expression of DVL2 and TGFB1 mRNA in Astrocytoma °II/°III biopsies (n = 10, *p* < 0.05), GBM biopsies (DVL: n = 17, *p* = 0.001; TGFB1: n = 39, *p* = 0.015), and primary GBM cell lines (n = 8, *p* < 0.001), as compared to normal brain tissue (n = 9). ** *p* < 0.001; * *p* < 0.05. n.s. = not significant.

To identify direct targets of miR-744 possibly accounting for the detected phenotypic alterations, we next combined in silico target prediction and pathway analysis to extract mRNAs (a) containing miR-744 binding sites in their 3’ untranslated region (3’UTR), and (b) being involved in the regulation of cellular mobility. This prompted us to investigate Transforming Growth Factor Beta 1 (TGFB1) and Dishevelled2 (DVL2) in detail, as these were the most promising predicted targets of miR-744 with respect to a supposed role in GBM migration [13,14]. To test this assumption, we quantified mRNA and protein expression levels of TGFB1 and DVL2 in U87 cells stably overexpressing miR-744, and indeed detected a marked decrease of TGFB1 and DVL2 mRNA (reduction of 38% ± 5.6% and 33.6% ± 4.9%, respectively, n = 5, *p* < 0.05), and protein expression (reduction of 35.6% ± 8.3% and 36.8% ± 5.3%; Figure 3A, left and middle panels). To provide experimental proof that both genes are bona fide targets of miR-744 in GBM, we performed luciferase reporter gene assays on vector constructs containing the full-length 3’-UTR of either TGFB1 or DVL2. As shown in Figure 3A (right panel), co-transfection of miR-744 mimic and reporter constructs diminished luciferase activity by 37% and 47%, respectively, compared to miR scrambled control (n = 5, *p* < 0.05), thereby proving that both genes are direct targets of miR-744. Transient knock-down of both TGFB1 and DVL2 by specific siRNAs (knock-down efficiency: Supplementary Figure S1) reduced the migratory capabilities of GBM cells in 2D migration assays (Figure 3B), thereby closing the anticipated functional loop; miR-744 impairs migration of human glioma cells by direct targeting of TGFB1 and DVL2.

In GBM and Astrocytoma °II/°III specimens, as well as in primary GBM cells, we found significantly increased expression levels of DVL2 and TGFB1. These results are in line with the detected down-regulation of miR-744 (Figure 3C; DVL2: GBM, induction 3.35-fold ± 0.14, *p* = 0.002; primary GBM cells, induction 5.4-fold ± 0.46, *p* < 0.001; normal brain tissue (NB), n = 9; GBM, n = 17; primary GBM cells, n = 8. TGFB1: GBM, induction 2.19-fold ± 0.09, *p* = 0.015; primary GBM cells, induction 4.33-fold ± 0.69, *p* < 0.001; NB, n = 9; GBM, n = 39; primary GBM cells, n = 8). Notably, GBM exhibited higher levels of TGFB1 and DVL2 as compared to Astrocytoma °II/°III (Figure 3; TGFB1: induction 1.5-fold ± 0.36, *p* = n.s.; DVL2: induction 2.1-fold ± 0.3, *p* < 0.001; n = 10 for Astrocytoma °II/°III).

Hence, this data indicates that miR-744 puts the brake on the expression of DVL2 and TGFB1, which both play an important role as promoters of migration in GBM.

### 2.4. Via Repression of DVL2 and TGFB1, miR-744 Regulates Beta-Catenin and SMAD-Signaling Pathways

**Figure 4:**
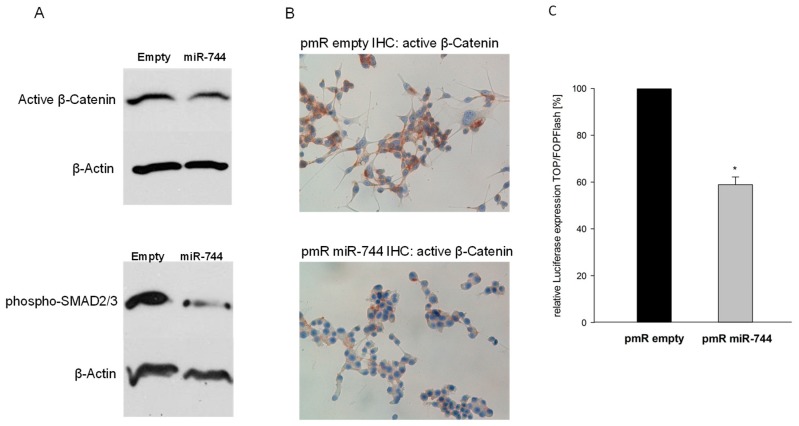
Stable overexpression of miR-744 represses activity of TGFB1 and DVL2 downstream effector molecules SMAD- and β-Catenin. Experiments were conducted after stable overexpression of miR-744 as compared to empty vector controls. (**A**) SDS-PAGE of active β-Catenin and phosphorylated SMAD 2/3. (**B**) Immunohistochemistry staining of active β-Catenin. (**C**) β-Catenin-dependent transcriptional activity of Lef/Tcf, measured with TOP/FOPFlash Luciferase Reporter Gene Assay (n = 4, *p* = 0.007). * *p* < 0.05.

We next set out to gain insight into the molecular pathways underlying the observed phenotypic alterations. TGFB1 is assumed to enhance cellular mobility through SMAD-dependent induction of pro-invasive factors such as Matrix Metalloproteinases (MMPs) [15,16], while DVL2 represents a central inducer of beta-Catenin signaling [17]. Both pathways induce epithelial-to-mesenchymal transition (EMT), and enhance tumor cell migration [14,17]. Consequently, we next assessed the activation status of SMAD2/3 and beta-Catenin upon overexpression of miR-744, as these transcription factors represent important downstream effector molecules of TGFB1 and DVL2 [16,18], respectively. As depicted in Figure 4A, miR-744 overexpression resulted in a marked reduction of the transcriptionally active forms of SMAD2/3 and beta-Catenin (SMAD2/3: reduction of 52% ± 15.8%; active beta-Catenin: reduction of 38.6% ± 5.7%). In addition, immunohistochemistry showed a significantly weaker staining for transcriptional active beta-Catenin upon overexpression of miR-744 as compared to controls (Figure 4B). In line with these results, subsequent analysis of beta-Catenin-mediated transcriptional activity by Lef/Tcf luciferase reporter gene assay revealed a significantly reduced activity in cells stably overexpressing miR-744 (Figure 4C; −46.1% ± 10.7%, n = 5, *p* < 0.01).

Taken together, our results show that miR-744 via DVL2 and TGFB1 ameliorates invasive properties of GBM cells by down-regulation of beta-Catenin and SMAD-signaling.

### 2.5. miR-744 Reduces It’s Host Gene MAP2K4 Through TGFB1-Mediated Negative Feedback

**Figure 5:**
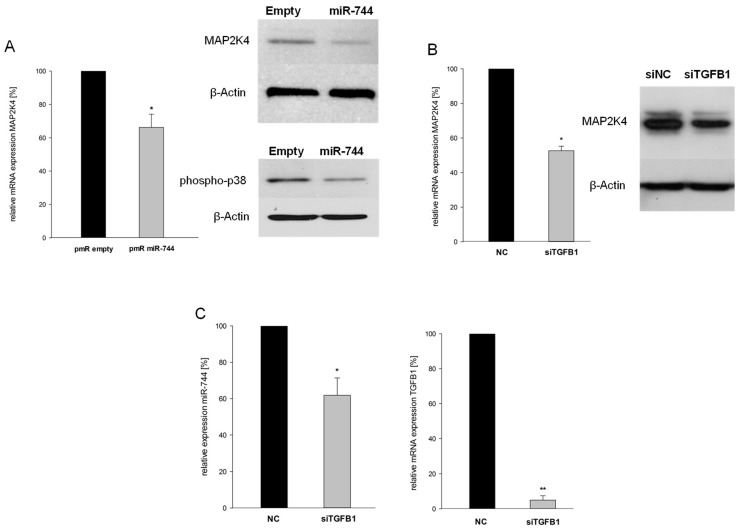
miR-744 reduces its host gene Mitogen-Activated Protein Kinase Kinase 4 (MAP2K4) through TGFB1-mediated negative feedback. (**A**) mRNA expression of MAP2K4 (left panel), and protein expression of MAP2K4 and its downstream target phospho-p38 MAPK (right panel), after stable overexpression of miR-744 as compared to empty vector controls (n = 5, *p* = 0.003). (**B**) mRNA (left panel) and protein expression (right panel) of MAP2K4 after knock-down of TGFB1 (n = 5, *p* = 0.004). (**C**) Expression of miR-744 (left panel) and TGFB1 (right panel) after knock-down of TGFB1 (miR-744: n = 5, *p* = 0.015; TGFB1: n = 5, *p* < 0.001). ** *p* < 0.001; * *p* < 0.05.

So far, our results have suggested that miR-744 acts as a counterpart of its host gene MAP2K4, an enhancer of cancer progression [8,10]. It thus appeared likely that a negative feedback relationship between miR and host might exist. To test this assumption, we assessed MAP2K4 expression in U87 cells stably overexpressing miR-744, and indeed found reduced mRNA and protein levels (Figure 5A; mRNA: −66.2% ± 7.9%; protein: −56.2% ± 9.6%; n = 5, *p* < 0.05). Concordantly, miR-744 overexpression attenuated activity of MAP2K4s downstream effector p38. As the 3’UTR of MAP2K4 does not contain any putative binding sites of miR-744, the host obviously is not directly targeted. We thus hypothesized that miR-744 indirectly represses its host by targeting an activator of the host gene. As TGFB1 is known to activate p38 MAPK, we speculated that TGFB1 might also fulfill a function as an enhancer of MAP2K4 signaling. Gene-specific knock-down of TGFB1 (Figure 5C, right panel) significantly decreased MAP2K4 mRNA and protein levels, thereby strongly supporting this assumption (Figure 5B; −47.7% ± 3.8%, n = 4, *p* = 0.004). Not unexpectedly, intronic miR-744 was also significantly affected by TGFB1 knock-down (Figure 5C, left panel; −38.1% ± 9.5%, n = 4, *p* = 0.015).

**Figure 6:**
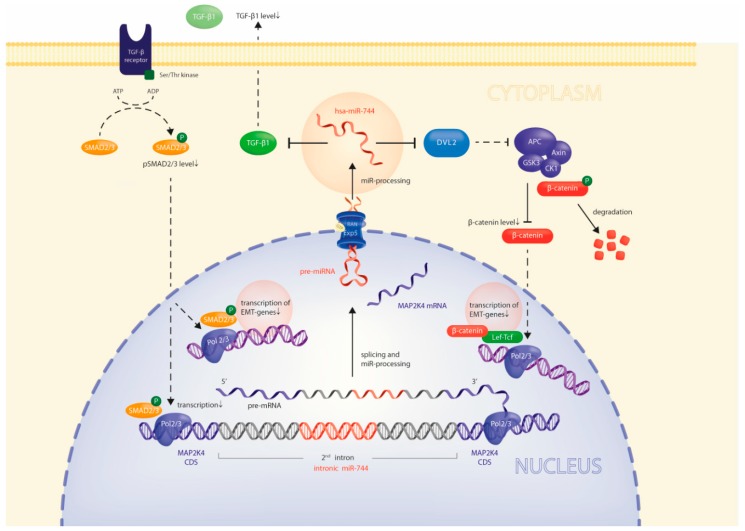
Model of miR-744 effects on it’s host gene´s expression and on tumor cell migration. Direct repression of DVL2 and TGFB1 by miR-744 inhibits SMAD- and beta-Catenin-dependent transcriptional activity, respectively, thus reducing expression of EMT- and pro-invasive genes. Moreover, miR-744 constitutes a second-order negative feedback loop on its host gene MAP2K4 through direct repression of TGFB1 levels, concomitantly repressing host gene and miR-744 expression.

These results sketch a regulatory loop: TGFB1 simultaneously enhances transcription of MAP2K4 and miR-744. The latter directly targets TGFB1, which as a negative feedback results in concurrent repression of MAP2K4 and miR-744 (Figure 6). These results highlight the close regulatory relationship between intronically located miRs and their host genes.

## 3. Discussion

In recent years, evidence has accumulated that colocalization of intronic miRNA and the host gene is not a random choice by nature, but rather fulfills important functional tasks within the host genes’ pathways. Several studies have experimentally proven in different contexts that intronic miRNAs are capable of either supporting or restraining functional pathways of their host genes, thereby creating intricate regulative networks [19,20]. As about half of human miRNAs reside in introns of protein-coding genes, these examples may signify a more general biological principle. A very recent study however, has suggested low prevalence of functional association between host and intronic miRNAs in general, but has assumed a key role of this type of regulation in cellular signaling pathways requiring tight control [21]. To date, experimental evidence supporting this view is scarce. As an example of potential high interest, we investigated miR-744, located in the second intron of the tumorigenic kinase MAP2K4, and its impact on it’s host gene’s functional networks.

Expression patterns of miRNAs are highly heterogeneous among cell types, and pronounced expression levels of a certain miRNA within a specific tissue is likely to reflect functional relevance. After analysis of different tissues, we found brain tissue to be the most suitable for further analyses, due to its high miR-744 expression. With respect to the role of MAP2K4 as a driver of malignancy, we assessed miR-744 expression in human brain tissue, in Astrocytoma WHO grade II/III, and in the most malignant brain tumor (i.e., GBM). Interestingly, we detected a gradual tumor-grade-dependent loss of expression, with almost completely lost miR-744 expression levels in GBM. These findings made us assume that miR-744 is a gatekeeper of oncogenic signaling during the malignization process. Indeed, stable re-expression of miR-744 in GBM cells resulted in a significantly more benign phenotype, with tumor cells growing focally, and migrating less. To uncover the mechanisms underlying these phenotype changes, we stably overexpressed miR-744 in GBM cells, and evaluated potential miR-744 target genes fulfilling the criteria of (a) being involved in the regulation of cell motility and (b) harboring potential miR-744 binding sites in their 3’UTRs. We identified and experimentally validated DVL2 and TGFB1 to be directly regulated by miR-744 in GBM cells. We were further able to show that specific knock-down of these target genes impaired cellular migration similar to miR-744 overexpression. In line with these results, we found that down-regulation of miR-744 in human GBM is accompanied by a marked increase in DVL2 and TGFB1 expression levels, indicating clinical relevance of these functional networks.

DVL2 and TGFB1 have repeatedly been shown to act as inductors of epithelial-to-mesenchymal transition (EMT) [13,22,23], one of the hallmarks of cancer progression. During EMT, induction of Matrix-Metalloproteinases initiates breakdown of the extracellular matrix and reduces cellular adhesion, which results in enhanced migratory capacity and invasiveness [24,25]. Particularly in GBM, TGFB1 is one of the most powerful cytokines secreted by the tumor itself. It has repeatedly been shown that GBM-derived TGFB1 induces EMT via activation of transcription factors of the SMAD family, thereby increasing migration of GBM cells into the surrounding brain tissue. It thus represents a hallmark of GBM progression [26,27,28]. In addition, DVL2, as a key enhancer of beta-Catenin transcriptional activity, represents another orchestrator of EMT [29,30,31]. In GBM, the DVL2-beta-catenin signaling axis has been found to be markedly activated, which significantly contributes to the extremely invasive nature of these tumors [13,32,33]. To this end, we tested whether inhibition of DVL2 and TGFB1 by overexpression of miR-744 exerted the supposed impact on the respective downstream effector molecules, beta-Catenin and SMAD. Based on immunohistochemistry, protein analyses, and reporter gene assays, our results provided evidence that miR-744 hampers GBM cell migration via concurrent reduction of SMAD- and beta-Catenin signaling. Thus, downregulation of miR-744 during gliomagenesis may be an effective tumor intrinsic mechanism to support tumor progression by simultaneously affecting different oncogenic signaling pathways.

This negative impact of miR-744 on tumor cell migration strongly contrasts with the functions of its host gene, MAP2K4, which has been shown to profoundly enhance cancer cell migration and metastasis [8,10]. It can thus be concluded that that miR-744 acts as functional antagonist of its host, thereby keeping a molecular balance. As stable overexpression of miR-744 resulted in significantly reduced MAP2K4 levels, we assumed that miR-744 may not only control its host functionally, but also on the level of gene expression through negative feedback. The concept of intronic miRs regulating their host genes by direct or indirect feedback loops has been experimentally proven in several different contexts [6,34]. Effects are achieved either directly by targeting of the host’s 3’UTR (first order negative feedback), or indirectly by targeting an interposed gene that subsequently affects the host gene’s expression (second order negative feedback). As the 3’UTR of MAP2K4 does not contain any predicted miR-744 binding sites, we focused on the identification of a second order negative feedback. In this regard, our finding that stable overexpression of miR-744 attenuated the activity of MAP2K4’s downstream effector p38 prompted us to come back to TGFB1 as an important activator of p38 MAPKs [35,36]. Indeed, we could show that gene-specific knock-down of TGFB1 significantly decreased MAP2K4 expression. Concomitantly, miR-744 levels were decreased, supporting the notion of a negative feedback loop acting as an “intrinsic transcriptional brake” to prevent inadequate transcription of MAP2K4. Inhibition of TGFB1 by miR-744 thus fulfills a dual role in mediating effects of this intronic miRNA on its host MAP2K4: (i) Functional antagonization via blocking SMAD-signaling, thereby reducing glioma migration and invasion; and (ii) control of the tumorigenic host’s expression levels.

It is a limitation of the current study that the molecular mechanisms enabling this fundamental switch remain elusive. However, due to the assumable multi-layered nature of these processes, this question may be addressed in further research projects.

## 4. Materials and Methods

### 4.1. Bioinformatics

Analysis of potential miR-mRNA interactions was performed using the public databases TargetScan, PITA, miRIAD, and picTAR. Potential direct interactions were considered probable when 2 or more algorithms returned a positive target prediction. In-silico analysis of miR expression levels was conducted using the intragenic microRNA database miRIAD (http://bmi.ana.med.uni-muenchen.de/miriad/). Involvement of target genes in tumor-associated pathways was evaluated using the KEGG database (www.genome.jp/kegg/pathway.html).

### 4.2. Human Tissue Samples

Tissue samples (n = 21, GBM; n = 15, WHO II/III Astocytoma; n = 8, primary GBM cell lines; and n = 9, normal brain) were obtained and processed as described previously [7]. Written informed consent was given by all patients, and the study protocol was approved by the Institutional Review Board of the Ludwig-Maximilians University of Munich, Germany (approval number: 216/14). For this study, only specimens from patients diagnosed with primary GBM, LOH 1p/19q negative, and IDH wild type were used.

### 4.3. RNA Extraction and cDNA Synthesis

RNA was extracted using the RNAqueous or miRvana Isolation kit (Ambion, Waltham, MA, USA), followed by DNAse treatment (Turbo DNAse, Ambion) according to the manufacturer’s instructions. RNA amount and quality was assessed using a NanoDrop 2000 spectrophotometer (Thermo Scientific, Waltham, MA, USA). Equal amounts of RNA were transcribed using Oligo-dT Primers, Random Hexamers (Qiagen, Venlo, Netherlands), dNTPs, RNAse OUT, and Superscript^®^ III Reverse Transcriptase (Invitrogen, Waltham, MA, USA), following the manufacturer’s instructions.

### 4.4. Quantitative RT-PCR

Quantitative analysis of mRNA levels was performed on a LightCycler 480 (Roche Diagnostics, Penzberg, Germany) using 10 ng of cDNA/well. Succinate Dehydrogenase Subunit A (SDHA) and TATA Box Binding Protein (TBP) were used as reference genes. Quantitative real-time PCR (qRT-PCR) was conducted using the primers (Metabion, Martinsried, Germany) and UPL Probes (Roche Diagnostics) provided in Supplementary Table S1. All assays were designed intron spanning. qRT-PCR conditions comprised initial denaturation for 10 Minutes (95 °C), and 50 cycles of 95 °C for 10 s, 60 °C for 30 s, and 72 °C for 1 s. Quantification cycle (C_q_) values were calculated employing the "second derivative maximum" method computed by the LightCycler^®^ software.

### 4.5. Quantification of miRNA Expression

Mir-744 expression was studied using TaqMan miRNA assays (Applied Biosystems, Waltham, MA, USA) according to manufacturer’s instructions. MiR-744 and reference gene expression was measured in technical duplicates. U47 served as an endogenous reference. All patient samples were calibrated using a sample of normal brain tissue.

### 4.6. SDS-PAGE

Cells were lysed in cell lysis buffer containing protease and phosphatase inhibitors (Cell Signaling Technologies, Danvers, MA, USA). Protein concentrations were assessed through BCA assays (Thermo Fisher Scientific, Waltham, MA, USA) according to the manufacturer’s instructions. Forty micrograms of the protein extracts were electrophoresed on 10% SDS-PAGE gels and electroblotted on PVDF-membranes. Nonspecific binding was blocked with 5% Bovine Serum Albumin (BSA) in TBS-Tween-20 (TBST) (Sigma, St.Louis, MO, USA). Antibodies for MAP2K4 (Cat. No. 9152), TGFB1 (Clone 56E4, Cat. No. 3709), DVL2 (Clone 30D2, Cat. No. 3224), and β-Actin (Clone 13E5, Cat. No. 4970) (all Cell Signaling Technologies) were diluted in TBST with 1% BSA. β-Actin served as the loading control. Immunoreactivity was assessed using horseradish peroxidase-labeled goat anti-mouse or goat anti-rabbit antibodies (Cell Signaling Technologies).

### 4.7. Cell Culture

U87 and T98G GBM cells were purchased from the American Type Cell Culture Collection (ATCC). Cells were maintained in DMEM (Gibco) with 10% FCS (Biochrom AG), 2% L-Glutamine, 1% Penicillin/Streptomycin, 1% MEM NEAA (Invitrogen), and 1% Sodium Pyruvate (PAA). HEK-293 cells (ATCC) were maintained in DMEM with 10% FCS, 2% L-Glutamine, 1% Penicillin/Streptomycin, and 1% MEM NEAA. Primary GBM cell lines were obtained from patients undergoing open GBM resection, according to the study protocol mentioned above. Tumor tissue was dissociated using the Brain Tumor Dissociation Kit P (Miltenyi, Bergisch-Gladbach, Germany) according to the manufacturer’s instructions. Primary GBM cells were cultivated in MACS Neuro Medium supplemented with Neuro Brew-21 without Vitamin A (Miltenyi).

### 4.8. Cloning of Reporter Constructs

The 3’UTRs of TGFB1 and DVL2 were amplified using genomic DNA and primer with XhoI and NotI, or PmEI and XhoI, restriction sites. Primer sequences are supplied in Supplementary Table S2. PCR products were cloned into the psiCHECK2 vector (Promega, Mannheim, Germany). Correct sequences were verified by Sanger sequencing (Eurofins Operon, Ebersberg, Germany). Plasmids were purified using the Qiaprep Spin Plasmid Miniprep Kit (Qiagen) and the Pure Yield Plasmid Midiprep System (Promega). DNA concentrations were determined using a NanoDrop 2000 spectrophotometer (Thermo Fisher Scientific).

### 4.9. Cloning of miRNA Expression Vector

The pre-miR sequence was amplified using genomic DNA and specific primers (sequences supplied in Supplementary Table S2). The resulting amplicon was cloned into the pmRZs-Green1 vector (Promega).

### 4.10. Transfections

Transfections were conducted using the NEON electroporation device (Life Technologies, Waltham, MA, USA). Transient transfection with microRNA precursors (premiR, Thermo Fisher Scientific) or siRNA (Dharmacon, Lafayette, CO, USA) was carried out at final concentrations of 50 nM (premiR) or 100 nM (siRNA), and 250,000 cells per well. Cells were incubated for 36 h at 37 °C and 5% CO_2_ in an antibiotics-free medium. Stable transfection was performed using 1 million cells and 10 µg plasmid/well. After incubation for 12 h in antibiotics-free medium, cells were seeded in DMEM containing 750 µg/mL Geneticin (Life Technologies). Stable transfection was analyzed by flow cytometry (Attune, Life Technologies). Monoclonal cell lines were obtained by single-cell picking. Overexpression of miR-744 was assessed through TaqMan assays. Co-transfection of luciferase reporter plasmids and premiR™ was carried out using 100,000 HEK-293 cells and 1 µg of Psi-CHECK™2 plasmid. All transfection experiments were performed in triplicate.

### 4.11. Reporter Gene Assays

After 36 hof incubation, co-transfected cells were harvested, washed twice, and resuspended in 20µl medium. Luminescence was measured with the MicroLumat Plus (Berthold Technologies, Bad Wildbad, Germany) using the Dual-Glo Luciferase Assay system (Promega), according to manufacturer’s instructions. All experiments were performed in triplicate.

### 4.12. Migration Assays

70,000 cells/well were seeded in 2-well culture inserts (Ibidi, Martinsried, Germany) and incubated for 24 h at 37 °C with 5% CO_2_ in a humidified incubator. Inserts were removed, cells were washed with cell culture media, and pictures were obtained using an inverted microscope (Carl Zeiss, Jena, Germany). Cells were incubated for 24 h in a cell culture incubator. Pictures were obtained and cells were fixed with methanol.Invasive properties were studied with the Cytoselect Assay, Collagen I, Colorimetric Format (Cell Biolabs, San Diego, CA, USA). 100.000 Cells were resuspended in 250 µL FCS-free media and seeded into the upper compartment of the boyden chamber. DMEM containing 10% FCS was added to the lower compartment. After incubation for 24 h in a cell culture incubator, non-invasive cells were removed with cotton swabs, invasive cells were stained, lysed, and the optical density was determined. Experiments were performed in duplicate.

### 4.13. Immunohistochemistry

100,000 cells were seeded on glass slides (Falcon) and incubated for 2 days. Cells were washed with PBS, fixed with ice-cold acetone, and washed with TBST. Endogenous peroxidase was blocked (1% H_2_O_2_, 10 minutes), cells were washed twice with TBST, and incubated in 5% normal goat serum for 1h. Antibodies were diluted in Antibody Diluent (Cell Signaling Technologies) at a concentration of 1:800 and incubated at 4 °C overnight. Cells were washed twice with TBST and Signal Stain Boost IHC Detection Reagent was added (Cell Signaling Technologies). After washing with TBST, Signal Stain DAB substrate was added for 45 s. Thereafter, cells were washed with TBST. Counterstaining was performed with Haematoxylin (Sigma Aldrich).

### 4.14. Statistics

All data is presented as the mean ± SEM. *p*-values were calculated using student’s *t*-tests. Statistical analyses were performed using SigmaPlot 12.0 (Systat Software). *p*-values below 0.05 were considered statistically significant (* *p* < 0.05; ** *p* < 0.001).

## 5. Conclusions

Taken together, we here uncovered a new regulatory circuit consisting of the tumorigenic host gene MAP2K4 and its intronically located miR-744; miR-744 acts as an intrinsic brake on its host by counterbalancing both its expression and function. In human glioma, this circuit is disrupted, leading to an invasion-promoting constellation where miR-744 is almost completely repressed while its host is induced.

Our data underscores the necessity to gain further profound insights into the networks of intronic miRNAs and their hosts, which is particularly important with respect to potential clinical approaches. Counteracting the shortfall of miR-744 in GBM pharmacologically, for example, might be an innovative clinical direction to pursue.

## Supplementary Materials

The following are available online at http://www.mdpi.com/2072-6694/10/11/400/s1, Table S1: Primer sequences for qRT-PCR; Table S2: Primer sequences for molecular cloning; Figure S1: Knock-down efficiency after transient transfection of U87 GBM cells with DVL2 or TGFB1 siRNA, as analyzed by qRT-PCR.
