# Supplementary material for: Intronic miR-744 Inhibits Glioblastoma Migration by Functionally Antagonizing Its Host Gene MAP2K4
Source: Cancers (Basel). 2018 Oct 25;10(11):400. doi: 10.3390/cancers10110400 (PMC6266622; doi:10.3390/cancers10110400)
Supplement: Supplementary file 1 [file cancers-10-00400-s001.pdf]

Supplementary Material

Intronic miR-744 Inhibits Glioblastoma Migration by Functionally Antagonizing Its Host Gene MAP2K4

Max Hübner, Christian Ludwig Hinske, David Effinger, Tingting Wu, Niklas Thon, Friedrich-Wilhelm Kreth and Simone Kreth

Table S1. Primer sequences for qRT-PCR.

Target Name	Primer Sequence	UPL Probe Number
TBP	forward: 5′-GAACATCATGGATCAGAACAACA-3′<br>reverse: 5′-ATAGGGATTCCGGGAGTCAT-3′	87
SDHA	forward: 5′-GAGGCAGGGTTTAATACAGCA-3′<br>reverse: 5′-CCAGTTGTCCTCCTCCATGT-3′	132
TGFB1	forward: 5′-ACTACTACGCCAAGGAGGTCA-3′<br>reverse: 5′-TGCTTGAACTTGTCATAGATTTCG-3′	33
MAP2K4	forward: 5′-GGCCAAAGTATAAAGAGCTTCTGA-3′<br>reverse: 5′-CAGCGATATCAATCGACATACAT-3′	31
DVL2	forward: 5′-GAGGAGGACACCATGAGC-3′<br>reverse: 5′-AAGGATGCGTCCTCTCC-3′	38

Table S2. Primer sequences for molecular cloning.

Gene	Primer Sequence
TGFB1	forward: 5′-GGTCCCGCCCCGCCCCGCCCC-3′<br>reverse: 5′-CCTCTCTCCATCTTTAATGGGG-3′
DVL2	forward: 5′-CAGTGGCAGTGAGTCTGAGC-3′<br>reverse: 5′-CAGCTACATGGCCCAAATCT-3′
miR-744	forward: 5′-AGATCTCTCCTTTGAGGGTGTTCTGTG–3′<br>reverse: 5′-CTGCAG CAGCATAAGCTTGCCAGGTA–3′

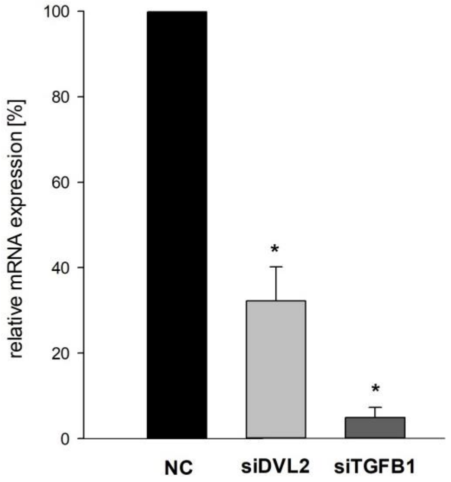

Figure S1. Knock-down efficiency after transient transfection of U87 GBM cells with DVL2 or TGFB1 siRNA, as analyzed by qRT-PCR (n = 3, * $p < 0.05$).

© 2018 by the authors. Licensee MDPI, Basel, Switzerland. This article is an open access article distributed under the terms and conditions of the Creative Commons Attribution (CC BY) license (http://creativecommons.org/licenses/by/4.0/).
